# A Nitro Functionalized MOF with Multi-Enzyme Mimetic Activities for the Colorimetric Sensing of Glucose at Neutral pH

**DOI:** 10.3390/s23146277

**Published:** 2023-07-10

**Authors:** Ya Wang, Yuanhua Wei, Siqi Li, Guang Hu

**Affiliations:** 1 Chongqing Key Laboratory of Medicinal Chemistry and Molecular Pharmacology, Chongqing University of Technology, Chongqing 400050, China; wyfance@163.com (Y.W.); wyhhhh0209@163.com (Y.W.); lisiqi47@cqut.edu.cn (S.L.); 2 Chongqing Institute of Innovation and Entrepreneurship for Precision Medicine, Chongqing 400050, China

**Keywords:** nanozyme, metal-organic frameworks, multi-enzyme mimetics, glucose, colorimetric sensor

## Abstract

Benefiting from the advantages like large surface area, flexible constitution, and diverse structure, metal-organic frameworks (MOFs) have been one of the most ideal candidates for nanozymes. In this study, a nitro-functionalized MOF, namely NO_2_-MIL-53(Cu), was synthesized. Multi-enzyme mimetic activities were discovered on this MOF, including peroxidase-like, oxidase-like, and laccase-like activity. Compared to the non-functional counterpart (MIL-53(Cu)), NO_2_-MIL-53(Cu) displayed superior enzyme mimetic activities, indicating a positive role of the nitro group in the MOF. Subsequently, the effects of reaction conditions on enzyme mimetic activities were investigated. Remarkably, NO_2_-MIL-53(Cu) exhibited excellent peroxidase-like activity even at neutral pH. Based on this finding, a simple colorimetric sensing platform was developed for the detection of H_2_O_2_ and glucose, respectively. The detection liner range for H_2_O_2_ is 1–800 μM with a detection limit of 0.69 μM. The detection liner range for glucose is linear range 0.5–300 μM with a detection limit of 2.6 μM. Therefore, this work not only provides an applicable colorimetric platform for glucose detection in a physiological environment, but also offers guidance for the rational design of efficient nanozymes with multi-enzyme mimetic activities.

## 1. Introduction

Nanozymes are a class of synthetic nanomaterials that exhibit enzyme-like catalytic activity. Compared with natural enzymes, nanozymes offer numerous advantages, including diverse structures, facile synthesis, high stability, and easy regulation and storage [1,2]. These characteristics have led to their widespread use in various fields, such as disease diagnosis, biosensing, environmental remediation, and so on [3,4,5]. Previous research has demonstrated that various nanomaterials can mimic different natural enzymes, including peroxidase [1,2,3], oxidase [4,5,6], superoxide dismutase [7], catalase, laccase, and others [8,9,10]. However, most of these studies have focused on the activity of single enzymes [6,7]. Recently, there has been a growing interest in multi-enzyme nanozymes, which can exhibit multi-enzyme mimetic activities alone or in combination, leading to synergistic effects, cascade reactions, and environmental response selectivity [8], as well as even broader application prospects. Despite the potential benefits, the development of highly efficient nanozymes with multiple activities is still in demand due to the limited number of nanozymes with well-defined structures, and multi-enzyme mimetic activities [9,10,11,12].

Metal-organic frameworks (MOFs) are porous crystalline materials composed of metal junctions and organic linkers [2]. Due to their large surface area, flexible constitution, and diverse structure, MOFs have emerged as ideal candidates for nanozymes [13,14]. Furthermore, the highly tunable and ordered structure of MOFs makes them attractive microreactors for dual-enzyme or multi-enzyme tandem-catalyzed reactions [15]. These characteristics suggest a great potential to construct nanozymes with multi-enzyme mimetic activities, which warrants further investigation.

Glucose, which is the crucial substance that produces energy in organisms, is very important for keeping the body functioning in good order [16,17]. Therefore, measuring blood glucose levels is essential for maintaining physiological health and treating diseases. Although there has been well-established research on glucose sensors, colorimetric techniques have gained high attention because of the development of the miniaturization concept, like paper—based analytical devices and image capture instruments [18]. Among the various colorimetric sensing modes, glucose oxidase (GOx)—based enzyme-catalyzed oxidation has proved to be a simple and reliable method for glucose detection. During the detection, glucose is hydrolyzed into gluconic acid, and H_2_O_2_ by GOx, and the in situ generated H_2_O_2_ is then catalyzed by peroxidase to oxidize the substrate for a visible color change. However, limited by the low catalytic efficiency, many previously reported peroxidase-like nanozyme to suffer from a low sensitivity for glucose detection. On the other hand, the instability of H_2_O_2_ and the difficulty of substrate oxidization under neutral pH have significantly hampered the development of this cascade reaction [19,20,21]. That means, most previous reports conducted the GOx incubation under neutral pH, and then, they had to adjust the pH into acid for peroxidase incubation. To overcome these shortcomings, it is still in demand to fabricate a novel peroxidase-like nanozyme with high catalytic efficiency under neutral pH, thus, constructing a simple and sensitive colorimetric platform for glucose detection.

**Figure 1:**
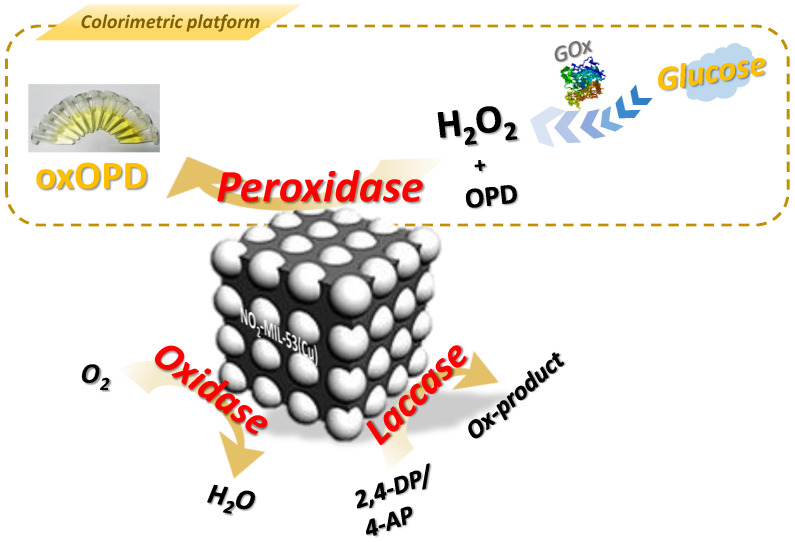
Triple-enzyme mimetic activities of NO_2_-MIL-53(Cu) and corresponding colorimetric platform for glucose detection via cascade reaction of GOx and its peroxidase-like activity.

In this work, we synthesized NO_2_-MIL-53(Cu) via a simple hydrothermal method. The regular complex structure centered on Cu allowed this material to mimic the activity of multiple enzymes, including peroxidase-like, oxidase-like, and laccase-like activity (as shown in Figure 1. To figure out the role of the functional group, MIL-53(Cu) was synthesized as a counterpart, and the activities were compared between NO_2_-MIL-53(Cu) and MIL-53(Cu). Then, the effects of different reaction conditions on enzyme mimetic activities were explored. Based on the excellent peroxidase-like activity of NO_2_-MIL-53(Cu) at neutral pH, a colorimetric sensing platform to detect H_2_O_2_ and glucose under a physiological environment was constructed.

## 2. Materials and Methods

All the reagents were of analytical grade and used as received without further purification. Copper (II) acetate monohydrate (Cu(CH_3_COO)_2_·H_2_O), nitroterephthalic acid (NO_2_-BDC), terephthalic acid (H_2_BDC), N,N-dimethylformamide(DMF), 2-(N-Morpholino)ethanesulfonic acid(MES), glucose and urea (CH_4_N_2_O) were purchased from Aladdin Co., Ltd. (Shanghai, China). Glucose oxidase (GOx) was provided by Sigma-Aldrich (Shanghai, China). o-phenylenediamine (OPD), 2,4-dichlorophenol (2,4-DP), and 4-aminoantipyrine (4-AP) were obtained from Macklin (Shanghai, China). Methanol was supplied by CHUANDONG Chemical (Chongqing, China). Milli-Q ultra-pure water with a resistivity of above 18.25 MΩ cm was used throughout the experiments.

The detection limits are calculated as LOD = 3σ/S, where σ was the standard deviation from 20 blank probe sample measurements, and s was the slope of the calibration plot from UV absorbance of solutions with different concentrations of targets (H_2_O_2_ or glucose).

### 2.1. Synthesis and Characterization of NO_2_-MIL-53(Cu)

The synthesis of NO_2_-MIL-53(Cu) was carried out using a modified version of a previously reported method [22]. A total of 0.6334 g of NO_2_-BDC and 0.7986 g of Cu(CH_3_COO)_2_·H_2_O were dissolved in 30 mL of H_2_O and stirred vigorously for 30 min. A total of 0.3903 g of urea was added to the mixed solution and stirred for another 30 min. The mixture was then transferred to a hydrothermal reactor with 50 mL of Teflon and subjected to solvothermal treatment at 150 °C for 5 h. After cooling to room temperature, the resulting precipitate was separated by using a 0.22 μm filter membrane and washed thoroughly with deionized water. Then, the collected product was washed with DMF and methanol several times. Finally, the obtained solid was dried at 60 °C for further characterization and application.

MIL-53(Cu) was synthesized by using 0.4984 g of H_2_BDC while keeping other steps unchanged.

The morphology and size of NO_2_-MIL-53(Cu) and MIL-53(Cu) were characterized by scanning electron microscopy (SEM, Gemini 300, Zeiss, Oberkochen, Germany). X-ray diffraction (XRD) patterns of the materials were measured with a scanning speed of 5°/min. Fourier transform infrared (FT-IR) spectra were acquired by PerkinElmer. Ultraviolet-visible (UV-vis) spectra were examined by a UV-2450 spectrophotometer (Unico, Shanghai, China).

### 2.2. Peroxidase-like Catalytic Feature Evaluation of NO_2_-MIL-53(Cu)

The peroxidase-like activity of NO_2_-MIL-53(Cu) nanozyme was measured by catalyzing the chromogenic substrate OPD with the presence of H_2_O_2_ at 37 °C. In detail, 100 μL NO_2_-MIL-53(Cu) suspension (0.2 mg mL^−1^), 30 μL H_2_O_2_ (10 mmol L^−1^), and 30 μL OPD (10 mmol L^−1^) solution were added in 840 μL Tris-HCl solution (0.01 M, pH 7.4), and incubated at 37 °C for 30 min. The final solution was analyzed via UV-vis spectrometer at 420 nm.

### 2.3. Colorimetric Detection of H_2_O_2_ and Glucose

For detection of H_2_O_2_, a series of H_2_O_2_ solutions with different concentrations over the range 0–1.4 mM were added to 100 μL of 200 mg/L NO_2_-MIL-53(Cu) suspension, and 30 μL OPD (10 mmol L^−1^) solution in Tris-HCl solution (0.01 M, pH 7.4). After incubation at 37 °C for 30 min, different UV absorption at 420 nm was recorded.

Glucose detection was as follows: firstly, 5 μL of Gox (1 mg/mL) and 20 μL of glucose solution with different concentrations were added to 115 µL of Tris-HCl buffer (0.01 M, pH 7.4), followed by incubation for 30 min at 37 °C. Subsequently, 100 μL NO_2_-MIL-53(Cu) suspension (0.2 mg/mL), OPD (10 mmol L^−1^), and Tris-HCl buffer (0.01 M, pH 7.4) were consecutively added to obtain a total volume of 1000 µL. The resulting mixture was incubated for another 30 min at 37 °C, and then measured by UV-vis spectrometer.

### 2.4. Oxidase-like Catalytic Feature Evaluation of NO_2_-MIL-53(Cu)

To explore the oxidase-mimicking behavior, 200 μL of NO_2_-MIL-53(Cu) suspension (0.2 mg mL^−1^) was added to an OPD solution (1 mM) in 0.01 M Tris-HCl buffer (pH 7.4) with a total volume 1 mL. The solution was also incubated at 37 °C for 30 min and finally monitored via UV-vis spectrometer.

### 2.5. Laccase-like Catalytic Feature Evaluation of NO_2_-MIL-53(Cu)

The laccase catalytic activity of NO_2_-MIL-53(Cu) nanozyme was measured through the chromogenic reaction of phenolic compounds with 4-AP. First, 4-AP (1 mg/mL, 100 µL) and 2,4-DP (1 mg/mL, 100 µL) solutions were mixed with MES buffer (30 mM, pH 6.8, 700 µL), followed by the addition of 100 µL NO_2_-MIL-53(Cu) suspension (1 mg/mL). After 1 h of reaction at 30 °C, the mixture was centrifuged for 3 min at 7000 rpm. Finally, the absorbance of the supernatant was recorded at 510 nm.

## 3. Results and Discussion

### 3.1. Synthesis and Characterization of NO_2_-MIL-53(Cu)

**Figure 2:**
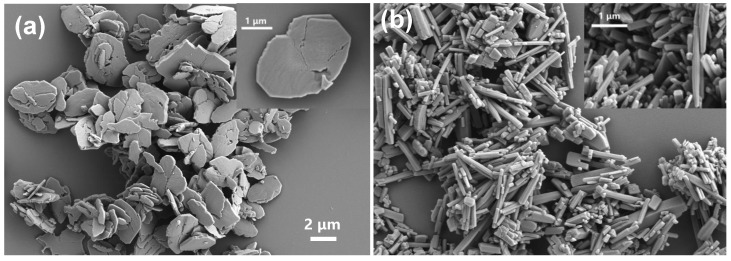
SEM images of (**a**) NO_2_-MIL-53(Cu), (**b**) MIL-53(Cu).

Previous research has demonstrated that the enzyme mimetic activity of nanozymes can be influenced by their functional groups [23]. In order to obtain nanozymes with superior activity, a nitro-functionalized MOF was synthesized according to the previous method with some modifications. As shown in Figure 2, the resulting NO_2_-MIL-53(Cu) exhibited a leaf-like structure with plan view sizes ranging from 2 to 4 μm (Figure 2a). In contrast, MIL-53(Cu), which was synthesized using the same method, displayed a stick-shaped structure with plan view sizes ranging from 0.2 to 1.5 μm (Figure 2b). The difference in morphology may be attributed to the addition of urea, a weak base used during the preparation process to deprotonate the groups in the organic linker and facilitate the coordination between metal ions and the organic linker. The protonation differences between BDC and NO_2_-BDC likely altered the kinetics of MOF formation [24,25]. 

**Figure 3:**
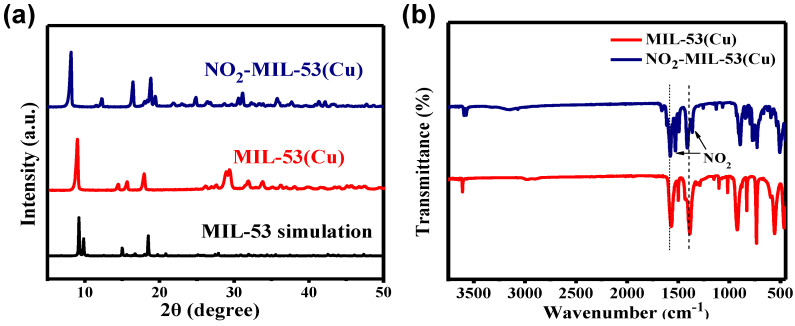
XRD patterns (**a**) and FT-IR (**b**) of NO_2_-MIL-53(Cu) and MIL-53(Cu).

Figure 3a shows the XRD patterns of NO_2_-MIL-53(Cu) and MIL-53(Cu). Both MOFs exhibited characteristic peaks similar to the MIL-53 simulation pattern, confirming their good crystalline structure [26,27]. The FT-IR spectra of NO_2_-MIL-53(Cu) and MIL-53(Cu) are shown in Figure 3b. Peaks near 1629 cm^−1^ and 1387 cm^−1^ represented the symmetric and asymmetric C=O stretching of COO^-^, respectively, indicating successful coordination between the BDC and the Cu ion [28]. Compared to MIL-53(Cu), NO_2_-MIL-53(Cu) displayed additional peaks in the region of 1510–1615 cm^−1^ and 1320–1390 cm^−1^, which corresponded to the antisymmetric stretching vibration of the nitro group. These characterizations provided further evidence of the successful synthesis of MIL-53(Cu) and NO_2_-MIL-53(Cu).

### 3.2. Multi-Enzyme Mimetic Activity of NO_2_-MIL-53(Cu)

**Figure 4:**
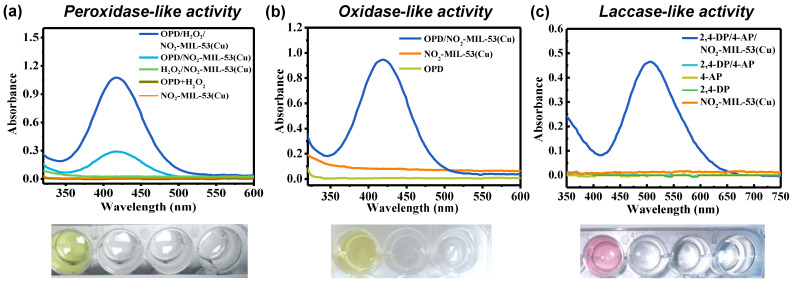
UV-vis spectra of (**a**) peroxidase-like, (**b**) oxidase-like, and (**c**) laccase-like activity of NO_2_-MIL-53(Cu) and corresponding images of different incubation systems (the solution in the image from left to right is consistent with the line from up to down; all of the images were taken under daylight).

To evaluate the multi-enzyme mimetic activity of NO_2_-MIL-53(Cu), various incubation systems were employed. The peroxidase-like activity of NO_2_-MIL-53(Cu) was assessed by oxidizing OPD in the presence of hydrogen peroxide in Tris-HCl (0.01 M) buffer. Figure 4a illustrates that strong absorbance and color changes (from colorless to yellow) were observed after NO_2_-MIL-53(Cu) was incubated with OPD/H_2_O_2_ for 30 min. In contrast, nearly negligible absorbance and color change were observed in the reaction systems of NO_2_-MIL-53(Cu)/OPD, NO_2_-MIL-53(Cu)/H_2_O_2_ or OPD/H_2_O_2_, indicating the excellent peroxidase-like activity of NO_2_-MIL-53(Cu). It should be noted that a small adsorption peak was also observed when NO_2_-MIL-53(Cu) reacted with OPD in the absence of H_2_O_2_, suggesting the oxidase-like activity of NO_2_-MIL-53(Cu). To confirm this oxidase-like activity, we re-tested the absorbance after incubating NO_2_-MIL-53(Cu) with OPD under a different reaction condition, in which the concentrations of catalyst and OPD increased into 40 mg/L and 1 mM, respectively (Figure 4b). These changes in concentration would promote the oxidase-like activity of NO_2_-MIL-53(Cu) via offered more active sites to initiate the redox reaction between oxygen and OPD. As expected, the solution presented a clear OPD oxidation peak at 420 nm, accompanied by a distinguishable color change from colorless to yellow. No peak was observed in the solution of NO_2_-MIL-53(Cu) and OPD alone, confirming the oxidase-like activity of NO_2_-MIL-53(Cu). Laccases were a family of copper-containing oxidases. To test the feasibility of NO_2_-MIL-53(Cu) as a laccase mimic, we incubated 2,4-dichlorophenol (2,4-DP) and 4-aminoantipyrine (4-AP) with NO_2_-MIL-53(Cu). As shown in Figure 4c, an absorption peak at 510 nm was observed after incubation, which can be attributed to the oxidation product of 2,4-DP. No absorption was observed with each compound alone. Similar results were observed in the image, in which only the solution with NO_2_-MIL-53(Cu) turned pink. The above results distinctly indicated that the prepared NO_2_-MIL-53(Cu) had laccase-like activity.

To uncover the role of the nitro group in the organic linker, MIL-53(Cu) was used as a non-functional counterpart to NO_2_-MIL-53(Cu). As shown in Figure S1, all of the incubation systems containing NO_2_-MIL-53(Cu) catalyzed substrates to produce high characteristic peaks, accompanied by obvious color change. In contrast, the systems containing MIL-53(Cu) only produced weak characteristic peaks and color changes. These results indicated that NO_2_-MIL-53(Cu) exhibited overwhelming enzyme mimetic activities, and the nitro group appears to play a positive role in the multi-enzyme mimetic catalysis. In previous reports by Wei et al., the nitro group was proposed as an electron-absorbing group that made the metal nodes in MOFs more electron-deficient, thereby promoting electron transfer between substrates and active sites [23]. This explanation could also be used to account for the improved activity of NO_2_-MIL-53(Cu).

### 3.3. Optimization of the Peroxidase-like Activity for NO_2_-MIL-53(Cu)

**Figure 5:**
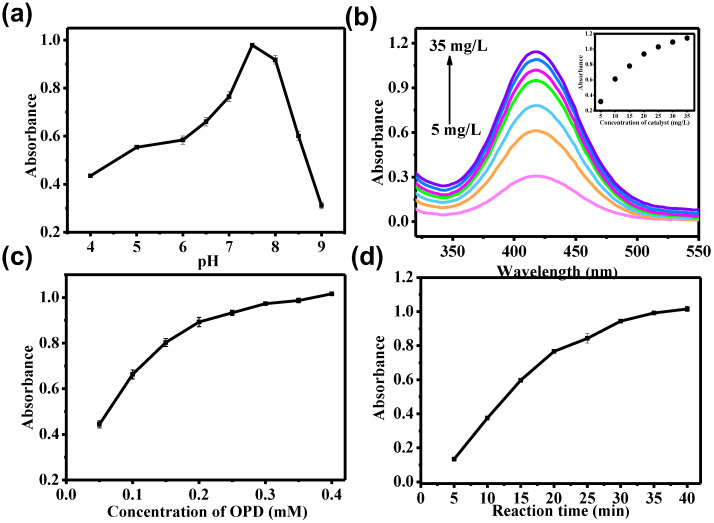
Effects of (**a**) pH, (**b**) catalyst concentration, (**c**) OPD concentration, and (**d**) reaction time on the peroxidase-like activity of NO_2_-MIL-53(Cu).

As it is known, the peroxidase-like activity of nanozyme is always dependent on reaction conditions. Therefore, the effects of typical conditions for NO_2_-MIL-53(Cu) were investigated. For solution pH, the peroxidase-like activity increased before pH 7.4 and decreased when pH exceeded 7.4 (as shown in Figure 5a), suggesting that the optimal pH for this reaction was around 7.4, which was consistent with the physiological environment. This applicability was further confirmed by testing different substrates. As shown in Figure S2, when we replaced OPD with other typical enzyme-linked chromogenic substrates, such as 3,3′,5,5′-tetramethylbenzidine (TMB), and 2,2′-azino-bis(3-ethylbenzothiazoline-6-sulfonic acid (ABTS), NO_2_-MIL-53(Cu) was able to catalyze the oxidation of different substrates under neutral conditions, demonstrating the wide applicability of peroxidase-like NO_2_-MIL-53(Cu).

To study the effect of catalyst concentrations, seven concentrations, including 5, 10, 15, 20, 25, 30, and 35 mg/L of NO_2_-MIL-53(Cu), were selected. As shown in Figure 5b, a growth trend was observed as the catalyst concentration increased, but the trend slowed down when the catalyst concentration exceeded 20 mg/L. 

We also explored the effects of OPD concentrations and reaction time, as shown in Figure 5c,d, respectively. When the OPD concentration increased to 0.3 mM, further increases in reaction concentration had little effect. A similar phenomenon was observed when the reaction time reached 30 min.

The stability of the peroxidase-like activity for NO_2_-MIL-53(Cu) was evaluated by storing NO_2_-MIL-53(Cu) in aqueous solution at 2–8 °C. Its peroxidase-like activity was measured for seven consecutive days. As shown in Figure S3, it was found that the activity of NO_2_-MIL-53(Cu) remained above 90% on the seventh day, indicating good stability.

### 3.4. Optimization of the Oxidase-like Activity and Laccase-like Activity for NO_2_-MIL-53(Cu)

Factors affecting the oxidase-like activity of NO_2_-MIL-53(Cu) were also investigated, including pH (ranging from 4 to 9), catalyst concentration (0, 5, 10, 15, 20, 25, 30, 35, 40, 45, 50 mg/L) and reaction time (5, 10, 15, 20, 25, 30, 35, 40, 45, 50 min). As shown in Figure S4a, NO_2_-MIL-53(Cu) exhibited high catalytic efficiency at pH values ranging from 7 to 8.5. With respect to reaction time, the absorbance of oxidized OPD increased with increasing reaction time and reached an appropriate UV measure range at 30 min (as shown in Figure S4b). Regarding the effect of catalyst concentration, the background absorbance from the catalyst should be taken into account. As shown in Figure S4c, the oxidase-like activity of NO_2_-MIL-53(Cu) reached stable when the catalyst concentration was up to 40 mg/L, since the differential value of absorbance tended to be constant at this concentration. 

In addition, we studied the effect of pH on the laccase-like activity of NO_2_-MIL-53(Cu). As shown in Figure S4d, NO_2_-MIL-53(Cu) exhibited the highest catalytic activity at pH 6.8, which was similar to natural laccase.

Nanomaterials with oxidase-like activity have been widely used for biosensors (such as the detection of metal ions and reducing substances), antibacterial and environmental protection [29,30,31]. As a green catalyst, nanomaterials with laccase-like activity have broad application prospects in environmental pollutant removal and environmental remediation [32,33]. In addition, laccase can also be used in biosensors (especially the detection of epinephrine), the food industry, and the paper industry [34,35,36]. Benefit from the synergistic effect of nanomaterials with multi-enzyme activity; they are also widely used in disease treatments. For example, Fe_3_O_4_/Ag/Bi_2_MoO_6_ nanoparticles with multiple-enzyme mimic activities are designed for multimodal imaging-guided chemical/photodynamic/photothermal therapy of tumors [37]. All of these examples have promised the great potential of NO2-MIL-53(Cu) in various fields.

### 3.5. Colorimetric Detection of H_2_O_2_ and Glucose

**Figure 6:**
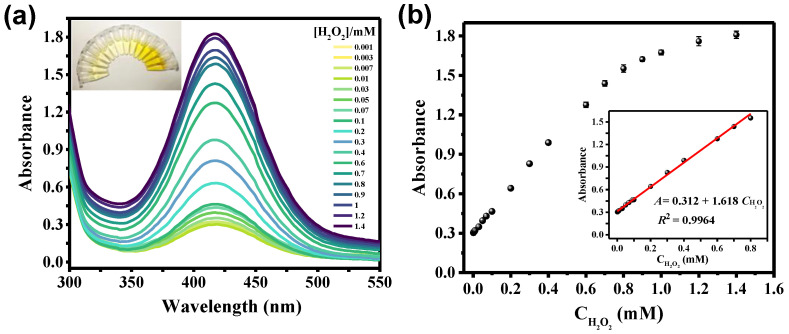
(**a**) UV-visible spectra of OPD/NO_2_-MIL-53(Cu) solution with different concentrations of H_2_O_2_ (inset is the corresponding colorimetric image, which was taken under daylight); (**b**) resulting calibration plots of the absorbance at 420 nm as a function of H_2_O_2_ concentration.

Based on the peroxidase-like activity of the NO_2_-MIL-53(Cu), we established a NO_2_-MIL-53(Cu)/OPD sensing system for colorimetric detection of H_2_O_2_. As shown in Figure 6, the color of the reaction solution gradually turned yellow upon the addition of H_2_O_2_ to MIL-53(Cu)/OPD. It was shown that the absorption intensity at 420 nm of the oxidized OPD increased sharply with increasing concentration of H_2_O_2_. A good linear relationship was found between the absorbance and H_2_O_2_ concentrations ranging from 1 to 800 μM (R^2^ = 0.9964). The limit of detection(LOD) for H_2_O_2_ was calculated as 0.69 μM (S/N = 3). 

As well known, glucose can be hydrolyzed by glucose oxidase(GOx) to produce H_2_O_2_ [38]. Therefore, a colorimetric platform can be achieved for glucose detection via the cascading reactions of NO_2_-MIL-53(Cu) and GOx. As shown in Figure S5, even in the incubation system of OPD/NO_2_-MIL-53(Cu), there was a weak absorption around 420 nm due to the inherent oxidase-like activity of NO_2_-MIL-53(Cu). This result is consistent with Figure 4a. When GOx or glucose was added separately, little change in this peak was observed. For the system containing GOx and glucose together, a significant increase in this peak can be observed. These results confirm that H_2_O_2_ is only produced when GOx is co-incubated with glucose, which in turn causes a peroxidase-like catalytic reaction.

**Figure 7:**
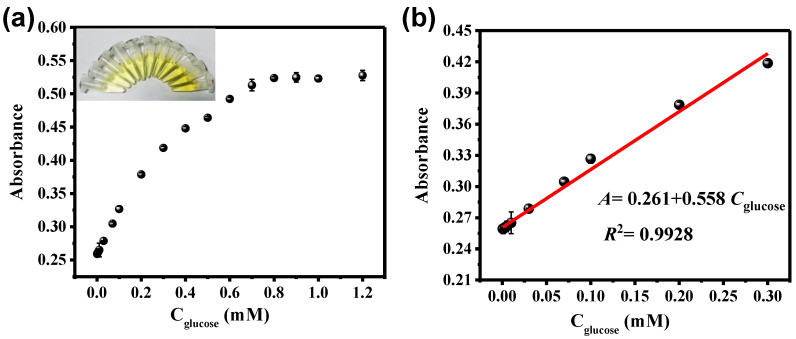
Dose-response curves and the corresponding linear calibration plots for glucose detection (**a**,**b**) (inset is the corresponding colorimetric image, which was taken under daylight).

Based on this, the absorbance at 420 nm increased with increasing glucose concentration (as shown in Figure 7a). Additionally, the inset image showed that the yellow color of the reaction system became darker with increasing glucose concentration. Figure 7b showed a good linear relationship between the glucose concentrations and the absorbance in the range of 0.5 μM to 300 μM (R^2^ = 0.9928), with a LOD of 2.6 μM (S/N = 3). This result is sufficient for the routine health screening of glucose in body fluids. Compared with previous reports, the performance of NO_2_-MIL-53(Cu) is also considerable (Table S1).

In order to evaluate the feasibility of the established platform for glucose detection, the effects of other sugars (sucrose, maltose, lactose, etc.) and common biochemical species in body fluids (amino acids, metal ions, ascorbic acid, etc.) were studied on the detection system. As shown in Figure S6, only the addition of the glucose triggered a significant chromogenic reaction, while the effect of other targets was negligible. These results demonstrated that the method has good selectivity for the detection of glucose.

## 4. Conclusions

In conclusion, we synthesized a nitro-functionalized MOF using hydrothermal methods. The synthesized NO_2_-MIL-53(Cu) demonstrated peroxidase-like, oxidase-like, and laccase-like catalytic activity. The effects of reaction conditions were discussed, and it was found that NO_2_-MIL-53(Cu) exhibited excellent peroxidase-like activity under neutral conditions. Based on this, a colorimetric sensing platform for hydrogen peroxide and glucose was constructed. Wide linear ranges could be achieved for H_2_O_2_ (1–800 μM) and glucose (0.5–300 μM), accompanied by low LODs of 0.69 μM for H_2_O_2_ and 2.6 μM for glucose, respectively. The platform exhibited good selectivity and promising potential feasibility for glucose detection in physiological environments. This work not only offered a more applicable colorimetric platform for glucose detection, but also provided guidelines for the rational design of nanozymes, especially for the materials with multi-enzyme mimetic activities.

## Data Availability

Not applicable.

## Supplementary Materials

The following supporting information can be downloaded at: https://www.mdpi.com/article/10.3390/s23146277/s1, Figure S1: UV-vis spectra and corresponding images of incubation systems with MIL-53(Cu) and NO_2_-MIL-53(Cu); Figure S2: UV-vis spectra and corresponding images of incubation systems in the presence of H_2_O_2_ with different enzyme-linked chromogenic substrates (a) TMB, (b) ABTS, (c) OPD; Figure S3: Peroxidase-like activity of NO_2_-MIL-53(Cu) during seven-day storing; Figure S4: Effects of (a) pH, (b) reaction time, (c) catalyst concentration on the oxidase-like activity of NO_2_-MIL-53(Cu). And (d) Effects of pH to the laccase-like activity of NO_2_-MIL-53(Cu); Figure S5: UV-vis spectra of different incubation systems in glucose detection; Figure S6: Effects of various common species towards glucose detection; Table S1: Comparison of various MOFs-based colorimetric detection platform for glucose detection. From refs. [39,40,41,42,43,44,45].
