# Supplementary material for: A Nitro Functionalized MOF with Multi-Enzyme Mimetic Activities for the Colorimetric Sensing of Glucose at Neutral pH
Source: Sensors (Basel). 2023 Jul 10;23(14):6277. doi: 10.3390/s23146277 (PMC10386029; doi:10.3390/s23146277)
Supplement: Supplementary file 1 [file sensors-23-06277-s001.zip › sensors-2460406-supplementary.pdf]

Supporting Information

A Nitro Functionalized MOF with Multi-enzyme Mimetic Activities for the Colorimetric Sensing of Glucose at Neutral pH

Ya Wang [1], Yuanhua Wei [1], Siqi Li [1,2], Guang Hu [1,*]

[1] Chongqing Key Laboratory of Medicinal Chemistry and Molecular Pharmacology, Chongqing University of Technology, Chongqing, China

[2] Chongqing Institute of Innovation and Entrepreneurship for Precision Medicine, Chongqing, China

* Correspondence: foxhu@cqut.edu.cn

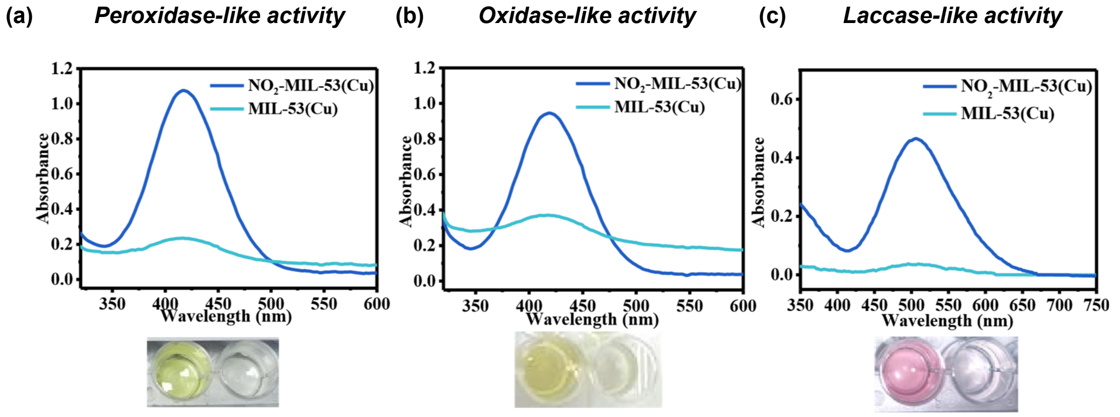

Figure S1 UV−vis spectra and corresponding images of incubation systems with MIL-53(Cu) and NO_2-MIL-53(Cu) (the solution in image from left to right is consist to the line from up to down and the images were taken under day light).

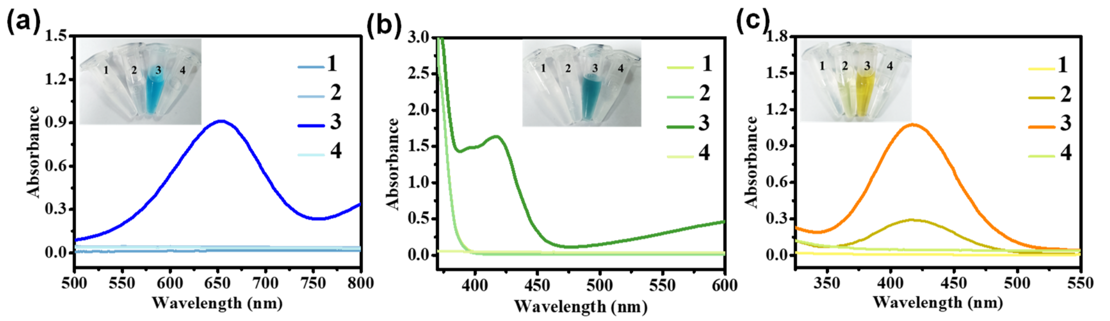

Figure S2 UV−vis spectra and corresponding images of incubation systems in the presence of H_2O_2 with different enzyme-linked chromogenic substrates (a) TMB; (b) ABTS; (c) OPD. (1-4 stand for different incubation systems, 1: chromogenic substrate/H_2O_2, 2: chromogenic substrate/ NO_2-MIL-53(Cu), 3: chromogenic substrate/ H_2O_2/NO_2-MIL-53(Cu), 4: H_2O_2/ NO_2-MIL-53(Cu), and the inset images were taken under day light).

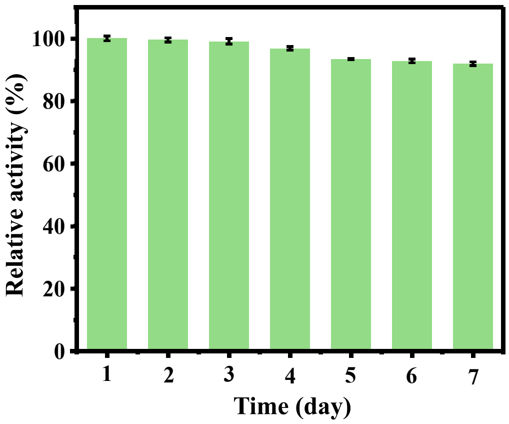

Figure S3 Peroxidase-like activity of NO_2-MIL-53(Cu) during seven-day storing.

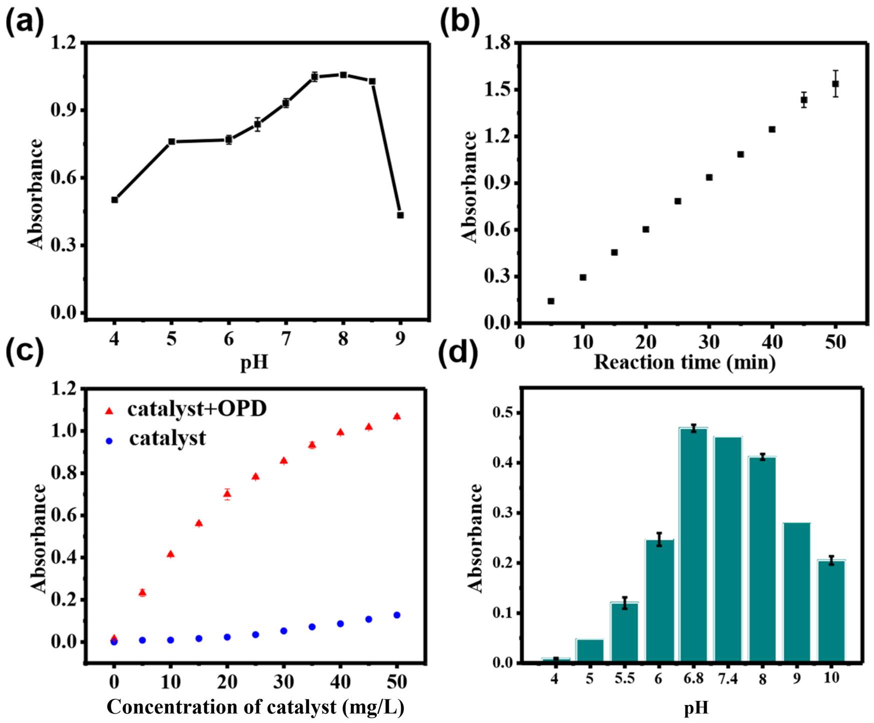

Figure S4 Effects of (**a**) pH, (**b**) reaction time, (**c**) catalyst concentration on the oxidase-like activity of NO_2-MIL-53(Cu). And (**d**) Effects of pH to the laccase-like activity of NO_2-MIL-53(Cu).

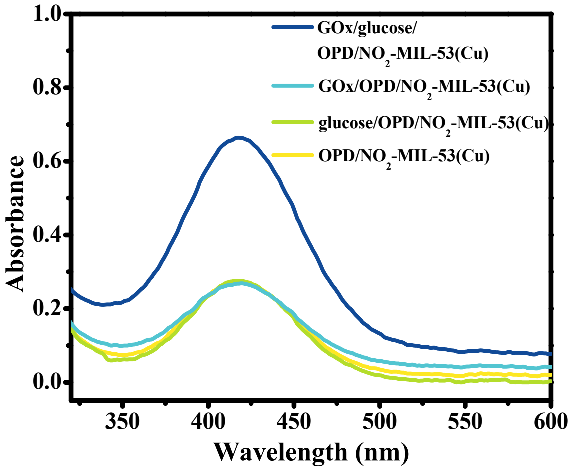

Figure S5 UV−vis spectra of different incubation systems in glucose detection (reaction concentrations: OPD 0.3 mM, GOx 5×10^{-3} mg/mL, glucose 2 mM, and catalyst 20 mg/L).

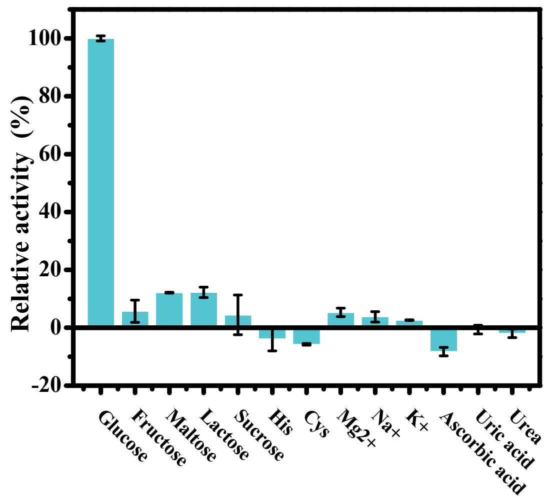

Figure S6 Effects of various common species towards glucose detection: (0.01 mM His and Cys; 0.02mM ascorbic acid and Uric acid; and the others 0.2 mM).

Table S1. Comparison of various MOFs-based colorimetric detection platform for glucose detection.

MOFs-based nanozymes	Peroxidase incubation pH	Linear range (μM)	LOD (μM)	Ref
Fe-BTC	4	5.0–100	2.4	[39]
Cu–hemin MOFs	6	10-3000	6.9	[40]
Magnetic ZIF-8	4	5-150	1.9	[41]
PCN-222(Fe)	4	12-75	2.2	[42]
CuBDC	5.2	10-500	4.1	[43]
Pt/Fe-MOF	4	2-400	2.3	[44]
Fe-MIL-101	7.4	up to 150	2.5	[45]
NO_2-MIL-53(Cu)	7.4	0.5-300	2.6	This work

References:

39. Zhao, Z.; Pang, J.; Liu, W.; Lin, T.; Ye, F.; Zhao, S., A bifunctional metal organic framework of type Fe(III)-BTC for cascade (enzymatic and enzyme-mimicking) colorimetric determination of glucose. *Microchim. Acta* **2019,** 186, (5).

40. Liu, F.; He, J.; Zeng, M.; Hao, J.; Guo, Q.; Song, Y.; Wang, L., Cu–hemin metal-organic frameworks with peroxidase-like activity as peroxidase mimics for colorimetric sensing of glucose. *J Nanopart Res* **2016,** 18, (5).

41. Hou, C.; Wang, Y.; Ding, Q.; Jiang, L.; Li, M.; Zhu, W.; Pan, D.; Zhu, H.; Liu, M., Facile synthesis of enzyme-embedded magnetic metal–organic frameworks as a reusable mimic multi-enzyme system: mimetic peroxidase properties and colorimetric sensor. *Nanoscale* **2015,** 7, (44), 18770-18779.

42. Aghayan, M.; Mahmoudi, A.; Nazari, K.; Dehghanpour, S.; Sohrabi, S.; Sazegar, M. R.; Mohammadian-Tabrizi, N., Fe(III) porphyrin metal–organic framework as an artificial enzyme mimics and its application in biosensing of glucose and H_2O_2. *J. Porous Mater.* **2019,** 26, (5), 1507-1521.

43. Cheng, X.; Zheng, Z.; Zhou, X.; Kuang, Q., Metal–organic framework as a compartmentalized integrated nanozyme reactor to enable high-performance cascade reactions for glucose detection. *ACS Sustain. Chem.Eng.* **2020,** 8, (48), 17783-17790.

44. Li, J.; Zhao, J.; Li, S.; Chen, Y.; Lv, W.; Zhang, J.; Zhang, L.; Zhang, Z.; Lu, X., Synergistic effect enhances the peroxidase-like activity in platinum nanoparticle-supported metal—organic framework hybrid nanozymes for ultrasensitive detection of glucose. *Nano Res.* **2021,** 14, (12), 4689-4695.

45. Ortiz-Gómez, I.; Salinas-Castillo, A.; García, A. G.; Álvarez-Bermejo, J. A.; de Orbe-Payá, I.; Rodríguez-Diéguez, A.; Capitán-Vallvey, L. F., Microfluidic paper-based device for colorimetric determination of glucose based on a metal-organic framework acting as peroxidase mimetic. *Microchim. Acta* **2017,** 185, (1).
